# Calcinosis Cutis With Selective Fibroblast Growth Factor Receptor Inhibitors: A Case Report and Review of Literature

**DOI:** 10.7759/cureus.83773

**Published:** 2025-05-09

**Authors:** Bipin Ghimire, Dhairya Gor, Omar Abbas, Maria Diab

**Affiliations:** 1 Hematology and Medical Oncology, Henry Ford Health System, Detroit, USA; 2 Pathology and Laboratory Medicine, Henry Ford Health System, Detroit, USA; 3 Internal Medicine, Michigan State University, Lansing, USA

**Keywords:** calcinosis cutis, calciphylaxis, fgfr inhibitor, hyperphosphatemia, pemigatinib

## Abstract

Selective fibroblast growth factor receptor (FGFR) inhibitors are emerging and promising treatment options in oncology, currently approved for metastatic cholangiocarcinoma and urothelial carcinoma. These agents are associated with various adverse events, including a range of dermatologic toxicities. In rare cases, they can cause calcinosis cutis (calcium deposition in the skin and subcutaneous tissue) or calciphylaxis (calcium deposition in blood vessels). Hyperphosphatemia, a common side effect, is considered a predisposing factor for these conditions. We report a rare case of pemigatinib-induced calcinosis cutis in a 46-year-old woman with FGFR2-TFAP2D fusion-positive metastatic cholangiocarcinoma. Ten days into treatment with pemigatinib, she developed painful, pruritic, erythematous leg lesions. Labs showed hyperphosphatemia with normal calcium; biopsy confirmed calcinosis cutis. Discontinuation of pemigatinib, phosphate binder therapy (calcium acetate), and topical steroids (triamcinolone) led to symptom resolution. We also review 10 similar cases linked to selective FGFR inhibitors, highlighting clinical features, management, and outcomes. Although rare, these conditions can be serious and potentially debilitating. Early recognition, regular phosphate monitoring, prompt intervention, drug adjustment or discontinuation, electrolyte correction, and wound care are key to improving patient outcomes.

## Introduction

Fibroblast growth factors (FGFs) are key heparin-bound proteins that play essential roles in various biological processes, including tumorigenesis [1]. They interact with four types of fibroblast growth factor receptors (FGFRs) in humans, namely, FGFR 1, 2, 3, and 4 [1].

FGFR signaling has been studied in cancers like biliary, urothelial, central nervous system (CNS), lung, and ovarian cancers, leading to the development of small molecule inhibitors against these receptors [2]. While several agents are under investigation, only a few selective FGFR inhibitors have received clinical approval in oncology. Erdafitinib was the first selective FGFR inhibitor approved for metastatic urothelial carcinoma; it acts as a pan-FGFR inhibitor (targeting FGFR 1-4) [2]. Similarly, futibatinib, also a pan-FGFR inhibitor, is approved for metastatic cholangiocarcinoma and gallbladder cancer [2]. Pemigatinib, indicated for metastatic cholangiocarcinoma and gallbladder cancer, selectively inhibits FGFR 1, 2, and 3 [2]. Infigratinib, another FGFR 1/2/3 inhibitor, initially received accelerated approval from the Food and Drug Administration for metastatic cholangiocarcinoma, but it was later withdrawn due to insufficient post-marketing trials demonstrating clinical benefit [3]. Other FGFR inhibitors, such as rogaratinib, derazantinib, and debio 1347, are currently under investigation [4].

A frequently reported adverse event associated with all selective FGFR inhibitors is hyperphosphatemia (60-85% of patients), which can lead to serious complications if not managed promptly [4,5]. In addition, dermatologic side effects are common, including dry mouth (29-59% of patients), dry skin (16-35%), alopecia (30-46%), and nail changes such as paronychia and onycholysis (30-35%) [4,5]. In rare cases, these therapies may cause calcium deposition disorders, like calcinosis cutis or calciphylaxis [4]. Most dermatologic side effects, excluding calcinosis cutis, are predominantly grade 1 or 2 and generally improve with supportive care or dose adjustments [4].

In this report, we present a case of calcinosis cutis following the initiation of pemigatinib and review the literature on other reported cases associated with selective FGFR inhibitors.

## Case presentation

A 46-year-old female initially presented with abdominal pain, nausea, and vomiting. Imaging revealed a large liver mass accompanied by a second liver mass and lymphadenopathy. She was diagnosed with metastatic cholangiocarcinoma after a liver mass biopsy. She began first-line chemoimmunotherapy, consisting of gemcitabine, cisplatin, and durvalumab. Notably, she had a history of psoriasis and was evaluated by dermatology prior to initiating immunotherapy.

**Figure 1:**
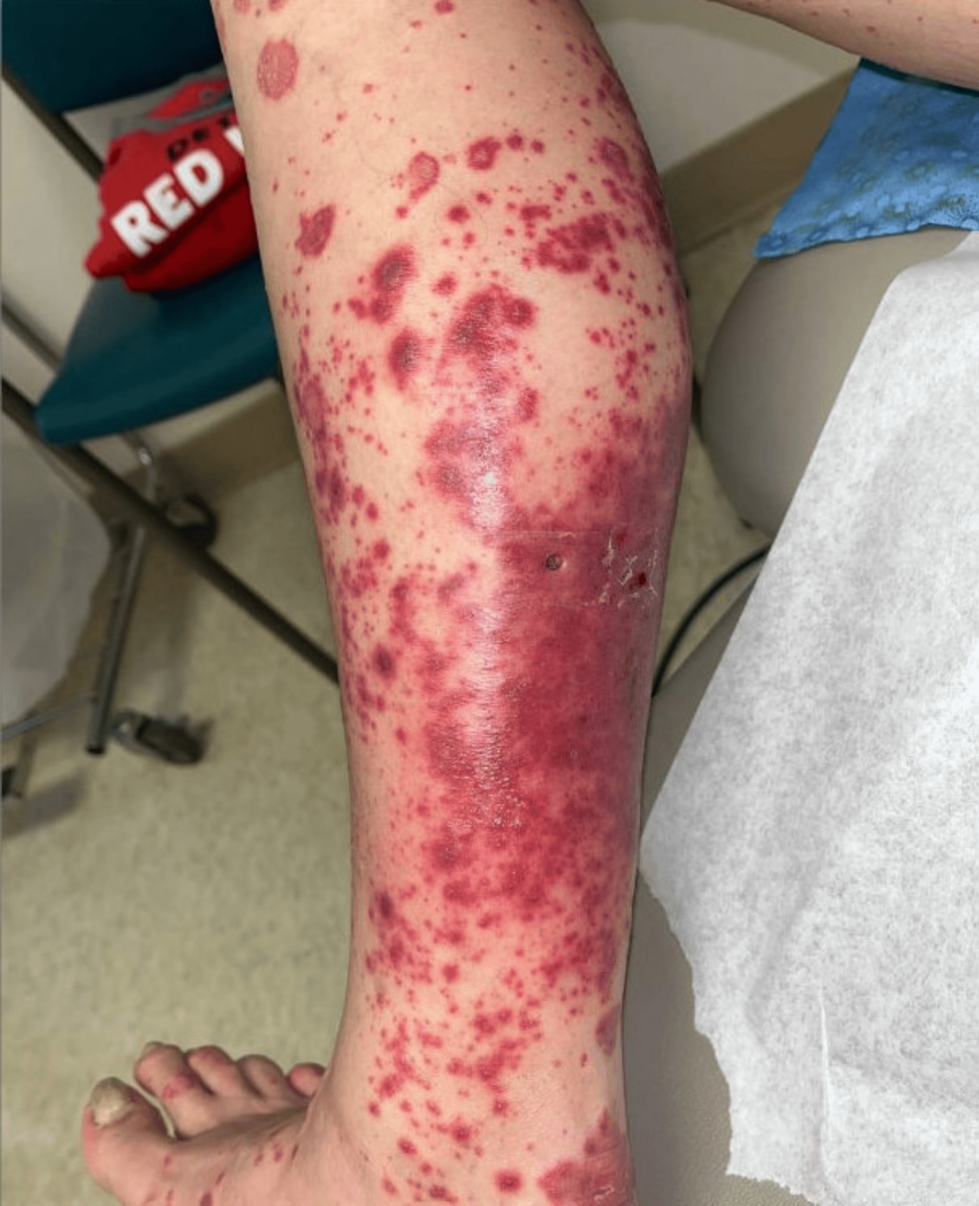
Skin lesion of the right lower extremity at onset

Unfortunately, after six months, disease progression was noted, and next-generation sequencing revealed an FGFR2-TFAP2D fusion, prompting the initiation of pemigatinib at the standard dose of 13.5 mg daily (14 days on, seven days off). Ten days into treatment, she developed painful, erythematous, and pruritic rashes on her legs, extending from below the knees to the ankles (Figure 1). She described the rash as distinct from her psoriatic lesions. The remainder of the physical examination was unremarkable. Laboratory tests revealed mild thrombocytopenia (platelets 135 x 10³/µL) with normal white blood cell counts and hemoglobin levels. A comprehensive metabolic panel, including renal and liver function tests, was normal; however, phosphorus was significantly elevated at 9.4 mg/dL, while calcium levels remained normal at 9.1 mg/dL. The patient’s laboratory values on presentation are listed in Table 1.

**Table 1:** Patient’s laboratory results on presentation

Laboratory parameters	Results	Normal reference range
White blood cell (WBC)	6.8 x 10^3^/µL	3.8-10.6
Hemoglobin	13.0 g/dl	12.0-15.0
Platelet	135 x 10^3^/µL	150-450
Sodium	137 mmol/L	135-145
Potassium	4.5 mmol/L	3.5-5.0
Blood urea nitrogen (BUN)	20 mg/dl	10-25
Creatinine	0.84 mg/dl	<1.16
Calcium	9.1 mg/dl	8.2 – 10.2
Phosphorus	9.4 mg/dl	2.5-4.5
Magnesium	2.0 mg/dl	1.8-2.3
Alanine aminotransferase (ALT)	7 IU/L	<52
Aspartate aminotransferase (AST)	14 IU/L	<35
Total bilirubin	0.9 mg/dl	<1.2
Alkaline phosphatase (ALP)	132 IU/L	40-140

**Figure 2:**
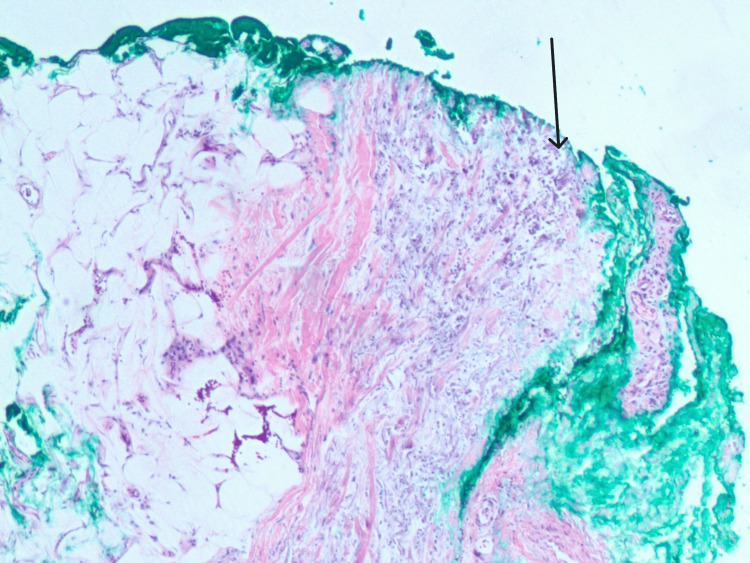
Histopathology from skin biopsy (H&E stain, 40x magnification): within the fat septae, seen are calcification of elastic fibers and rare calcified vessels in the fat

**Figure 3:**
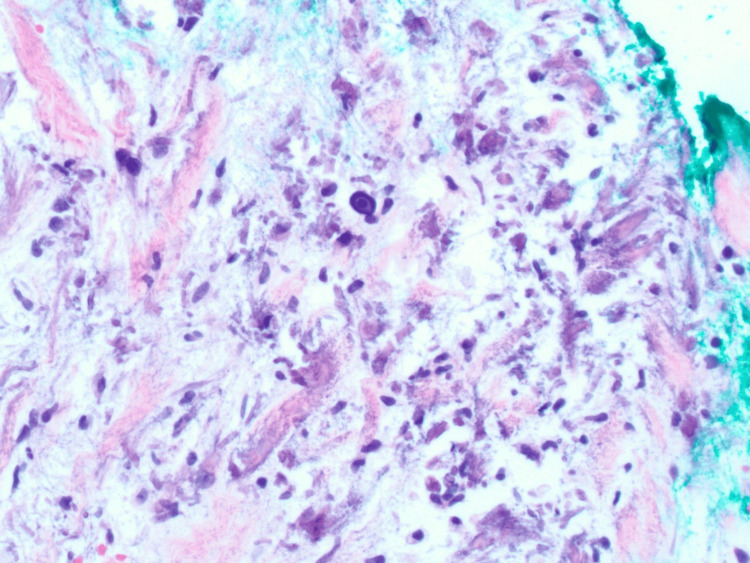
Histopathology from skin biopsy (H&E stain, 200x magnification): higher magnification showing calcified elastic fibers and calcium deposits

**Figure 4:**
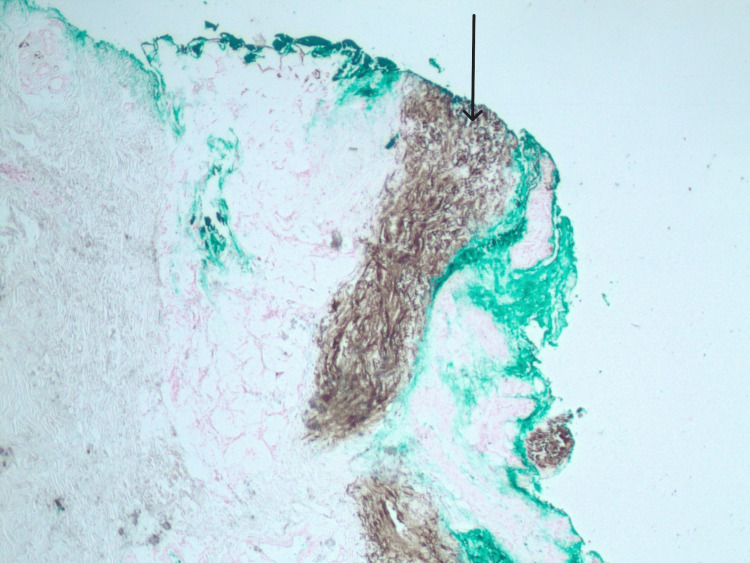
Histopathology from skin biopsy (Von kossa stain, 20x magnification): von kossa Stain highlighting calcification of elastic fibers

A skin punch biopsy demonstrated focal vascular calcification with elastic fiber calcification, suggesting a diagnosis of either calciphylaxis or calcinosis cutis (Figures 2, 3, 4). The treating team deemed the clinical findings atypical for calciphylaxis and planned further follow-up. Meanwhile, pemigatinib was temporarily held, and she was started on a phosphate binder (calcium acetate 667 mg three times daily) and topical steroid (triamcinolone 0.1% twice daily). Ten days after the initiation of calcium acetate, her phosphorus level normalized (3.4 mg/dl).

A second skin biopsy two weeks later revealed significant calcium deposition in the deep dermis and subcutaneous tissue, with relative sparing of the blood vessels, alongside changes indicative of psoriasis and leukocytoclastic vasculitis. Treatment with topical triamcinolone was continued. Given concerns regarding worsening calcinosis cutis if pemigatinib were continued, treatment was switched to 5-fluorouracil and liposomal irinotecan. Discontinuation of pemigatinib and normalized phosphorus level allowed for the cessation of the phosphate binder. On subsequent follow-up, her skin lesions had completely resolved.

## Discussion

Calcinosis cutis and calciphylaxis

Calcinosis cutis is defined as the deposition of calcium salts in the skin and subcutaneous tissue, often due to dystrophic calcification, which occurs in response to tissue damage from systemic or localized diseases [6]. In these cases, serum calcium and phosphorus levels are typically normal [6]. On the other hand, metastatic calcification is linked to abnormal calcium and phosphorus level, particularly when the calcium-phosphate product exceeds 70 mg²/dL² [6].

Calciphylaxis, a severe manifestation of calcinosis, involves calcification of small and medium-sized vessels and is commonly linked to chronic renal failure and dialysis [7]. However, non-uremic calciphylaxis has also been reported. A systematic review by Nigwekar et al. identified primary hyperparathyroidism, malignancies, alcoholic liver disease, and connective tissue diseases as common predisposing factors for non-uremic calciphylaxis [7].

Mechanism of calcium deposition with FGFR inhibitors

The mechanism underlying calcium deposition associated with FGFR inhibitors remains unclear [4,8]. While hyperphosphatemia, leading to an increased calcium-phosphate product, was initially thought to be the primary cause of calcinosis, there are cases reported with normal calcium and phosphate levels [8]. Increased expression of the FGF2 receptor at sites of calcification in atherosclerotic plaques suggests that FGFR inhibition may contribute to vascular calcification [4]. FGFR inhibitors may also induce a compensatory increase in FGF-23, a hormone involved in phosphate regulation, potentially promoting vascular calcification independent of phosphate levels [8]. Here, we summarize the clinical characteristics of patients experiencing calcinosis cutis or calciphylaxis secondary to selective FGFR inhibitors.

Calcinosis cutis associated with selective FGFR inhibitors

Despite the range of dermatologic adverse events linked to FGFR inhibitors, reports of calcinosis cutis or calciphylaxis remain rare. Our literature review, including our case, identified 11 instances of selective FGFR inhibitor-induced calcinosis cutis or calciphylaxis. The clinical characteristics of these patients are summarized in Table 2. Seven cases were associated with pemigatinib [8-13], two with erdafitinib [14,15], one with infigratinib [16], and one with a pan-EGFR inhibitor (generic or scientific name not specified) [17]. Notably, no cases were linked to futibatinib, consistent with a safety analysis of 469 patients in phase I and II studies that did not report any cases of calcinosis cutis [18].

**Table 2:** Clinical characteristics of patients with selective FGFR inhibitor-induced calcinosis cutis Abbreviations: B/L: bilateral; FGFR: fibroblast growth factor receptor; IV: intravenous; NA: not available; Phos: phosphorus

Medication	Indication	Age/sex	Site of calcinosis	Calcinosis onset	Pathologic diagnosis	Highest phos level (mg/dl)	FGFR inhibitor treatment	Other intervention	Calcinosis outcome	Reference
Pemigatinib	Cholangiocarcinoma	82/F	B/L legs	3 months	Calcinosis cutis and calciphylaxis	6.2	Discontinued	IV sodium thiosulfate	Improved	[8]
Pemigatinib	Wilm’s tumor	24/M	B/L axilla, B/L popliteal fossa	6 weeks	Calcinosis cutis	7.9	Discontinued	NA	NA	[9]
Pemigatinib	Cholangiocarcinoma	50/M	B/L thighs	12 days	Calcinosis cutis and calciphylaxis	7.5	Discontinued	IV and topical sodium thiosulfate	Improved	[10]
Pemigatinib	Cholangiocarcinoma	48/F	B/L legs, B/L axilla	5 weeks	Calcinosis cutis	8.7	Continued	Phosphate binder	Resolved	[11]
Pemigatinib	Cholangiocarcinoma	59/F	Back, B/L hips, legs	4 months	Calcinosis cutis	7.5	Discontinued	Phosphate binder	Improved	[12]
Pemigatinib	Pancreatic adenocarcinoma	62/M	B/L thighs and B/L axilla	25 days	Calcinosis cutis	6.4	Drug dose reduction	IV sodium thiosulfate	Improved	[13]
Pemigatinib	Cholangiocarcinoma	46/F	B/L legs	10 days	Calcinosis cutis	9.4	Discontinued	Phosphate binder	Resolved	Present case
Erdafitinib	Urothelial carcinoma	70s/F	B/L thighs	18 days	Calcinosis cutis and calciphylaxis	5.6	Discontinued	NA	Resolved	[14]
Erdafatinib	Uterine adenosarcoma	50s/F	B/L thighs, legs	3 months	Calcinosis cutis and calciphylaxis	3.8	Discontinued	Topical nitroglycerin	NA	[15]
Infigratinib	Cholangiocarcinoma	60/M	B/L axilla, B/L inguinal folds	8 weeks	Calcinosis cutis	8.6	Discontinued	Prophylactic phosphate binder before treatment	Not reported	[16]
Pan FGFR inhibitor (in clinical trial)	Urothelial carcinoma	69/M	B/L axilla, digital ulcers	3 months	Calcinosis cutis	7.3	Discontinued	NA	Resolved	[17]

The 11 patients ranged in age from 24 to 82 years at the onset of skin lesions, with six females and five males. The most common sites of calcinosis were the lower extremities (in nine patients), while five had lesions in the axilla, and three exhibited lesions in both locations.

Histopathology revealed calciphylaxis in four patients, while the remaining had calcium deposits solely in the skin or subcutaneous tissue. The onset of skin lesions ranged from 10 days to four months after starting EGFR inhibitors, with the quickest onset observed in our case. Ten patients exhibited hyperphosphatemia at diagnosis; in one patient, phosphorus levels were reported only after treatment discontinuation.

Following the diagnosis of calcinosis cutis, the FGFR inhibitor was discontinued in most cases (nine of 11). One patient resumed treatment with a reduced dose after a brief interruption, while another continued the medication. Both patients were receiving pemigatinib and were able to continue it along with a phosphate binder without worsening or recurrence of skin lesions. In addition to discontinuation or dose reduction of the FGFR inhibitor, three patients received sodium thiosulfate, and three were treated with oral phosphate binders as adjunct therapies. Most patients experienced an improvement in their skin lesions post-intervention, although outcomes were not reported for two cases that experienced systemic disease progression.

Management options

Hyperphosphatemia is a common adverse event associated with FGFR inhibitors, occurring in 60-85% of clinical trial patients, with most cases being grade 1-2 in severity [5]. Early management of hyperphosphatemia could prevent the development of calcinosis cutis. Serum phosphate levels should be monitored regularly during FGFR inhibitor treatment [5]. Management options include dietary restrictions, phosphate-lowering therapies (e.g., calcium carbonate, sevelamer hydrochloride), and possible adjustments to FGFR inhibitor dosing [5].

Treating calcinosis cutis or calciphylaxis requires a multidisciplinary approach, involving adjusting the offending agents, correcting electrolyte imbalances, wound care, and pain management [19]. Intravenous or intralesional sodium thiosulfate is commonly employed for uremic calciphylaxis, although its precise mechanism remains unclear [4,19]. Despite uncertain efficacy, it is also considered for non-uremic calciphylaxis, as noted in the cases described above. Other treatment modalities, such as diltiazem, hyperbaric oxygen therapy, and bisphosphonates, have also been proposed for managing calcinosis cutis and calciphylaxis [6,19].

## Conclusions

Calcinosis cutis has been rarely reported with selective FGFR inhibitors, but it can be debilitating for patients. Hyperphosphatemia is considered a common predisposing factor for the development of calcinosis cutis, highlighting the importance of regular monitoring and management of phosphate levels during treatment with FGFR inhibitors to prevent this condition.

We recommend maintaining a high index of suspicion for calcinosis cutis in patients experiencing new skin lesions or related symptoms while on selective FGFR inhibitor therapy. Early detection, combined with a multidisciplinary treatment approach, is crucial to prevent complications and reduce the morbidity associated with calcinosis cutis and calciphylaxis. Adjustment or discontinuation of the offending agent, along with correction of electrolyte disturbances and appropriate wound care, will likely lead to clinical improvement.
